# *Piper nigrum*, *Piper betle* and *Gnetum gnemon*- Natural Food Sources with Anti-Quorum Sensing Properties

**DOI:** 10.3390/s130303975

**Published:** 2013-03-20

**Authors:** Li Ying Tan, Wai-Fong Yin, Kok-Gan Chan

**Affiliations:** Division of Genetics and Molecular Biology, Institute of Biological Sciences, Faculty of Science, University of Malaya, Kuala Lumpur 50603, Malaysia; E-Mails: etaly87@yahoo.com (L.Y.T.); yinwaifong@yahoo.com (W.-F.Y.)

**Keywords:** natural products, *Pseudomonas aeruginosa*, pathogenicity, pyocyanin, quorum sensing, quorum quenching, swarming, violacein, virulence

## Abstract

Various parts of *Piper nigrum, Piper betle* and *Gnetum gnemon* are used as food sources by Malaysians. The purpose of this study is to examine the anti-quorum sensing (anti-QS) properties of *P. nigrum*, *P. betle* and *G. gnemon* extracts. The hexane, chloroform and methanol extracts of these plants were assessed in bioassays involving *Pseudomonas aeruginosa* PA01, *Escherichia coli* [pSB401], *E. coli* [pSB1075] and *Chromobacterium violaceum* CV026. It was found that the extracts of these three plants have anti-QS ability. Interestingly, the hexane, chloroform and methanol extracts from *P. betle* showed the most potent anti-QS activity as judged by the bioassays. Since there is a variety of plants that serve as food sources in Malaysia that have yet to be tested for anti-QS activity, future work should focus on identification of these plants and isolation of the anti-QS compounds.

## 1. Introduction

Quorum sensing (QS) is a widespread, well-known cell-to-cell communication phenomenon in proteobacteria. QS is used by proteobacteria for the regulation of behaviors such as bioluminescence, biofilm formation, antibiotic production, conjugation and virulence [1–3]. QS comprises of chemical communication among bacteria involving formation, secretion, detection and reaction to molecules known as autoinducers (AI). When the concentration of the AI in the population has reached a certain threshold level (which happens when bacteria cell density is high), the gene expression of the bacteria is altered as a respond to this event [1]. AIs or signaling molecules used by Gram-negative bacteria are known as *N*-acyl homoserine lactones (AHLs), while Gram-positive bacteria utilize post-translationally modified oligopeptides as signalling molecules [4].

*Chromobacterium violaceum* was first reported as a pathological strain when studies showed that this bacterium is the cause of infections in fetal water buffaloes in the Philippines [5]. *C. violaceum* is commonly found in soil and water, particularly in the tropical and subtropical areas and produces violacein, a purple pigmented compound when *N*-hexanoylhomoserine lactone (C6-HSL) is present [6,7]. *C. violaceum* CV026 is a mini-Tn*5* mutant of *C. violaceum* which does not produce violacein unless it is supplied with C6-HSL. *C. violaceum* CV026 lacks the autoinducer synthase CviI and thus requires exogenous C6-HSL for violacein formation, which is QS-mediated [8].

On the other hand, *Pseudomonas aeruginosa* is an opportunistic pathogenic bacterium which is best known for its destructive effects in cystic fibrosis patients [9]. QS plays a major role in the regulation of *P. aeruginosa* virulence expression such as biofilm, pyocyanin, elastase, swarming and protease [10]. *P. aeruginosa* have two distinct yet hierarchal QS circuits, namely LasI-LasR and RhlI-RhlR. LasI, which is a LuxI homologue, produces 3-oxododecanoylhomoserine lactone (3-oxo-C12-HSL) that binds to LasR. Then, the LasR-autoinducer complex activates a range of genes including *lasI*, and a positive feedback loop from this interaction further activates the system [11,12].

Since it was known that the virulence phenotypes of bacteria can be quenched by blocking the QS, ongoing current research has been dedicated to the search for anti-QS compounds [13–23]. Anti-QS effects can be achieved by enzymatic approaches or using natural products [13]. Many quorum quenching bacteria producing AHL degradation enzymes have been isolated and studied [14–17]. Recent studies have demonstrated that QS antagonist compounds can be found in higher plants such as peas, vanilla, raspberry, *Melicope lunu-ankenda*, clove, and *Myristica cinnamomea* [18–23]. Our group has reported previously that malabaricone C isolated from the *Myristica cinnamomea* methanolic extract shows anti-QS activity that inhibits the virulence determinants of *P. aeruginosa* PAO1 [23]. In light of this finding, we have performed a systematic screening on edible plants in Malaysia in search of compounds with anti-QS properties.

*Piper nigrum* (common name: peppercorn) is a natural spice widely used in the Ayurvedic medicine. It is used in treatment for asthma, cough, diabetes and heart problems [24]. On the other hand, *Piper betle* (common name: betle leaves) was shown to contain compounds that have anti-diabetic and anti-allergic effects [25,26]. *Gnetum gnemon* (common name: belinjo leaves) fruits and leaves are consumed in Southeast Asians countries and the study conducted by Kato *et al.* [27] found that the seeds of *G. gnemon* have anti-oxidant properties.

In this study, we assessed the anti-QS properties of *P. nigrum*, *P. betle* and *G. gnemon* against *P. aeruginosa* PAO1, *C. violaceum* CV026 and *Escherichia coli* [pSB 401] and *E. coli* [pSB1075]. We found that the extracts of these plants possesses anti-QS properties and future studies should involve identification of the active compound(s) and the mechanism of action.

## 2. Experimental Section

### 2.1. Plant Sample Identification, Deposition of Voucher Specimens and Preparation of Plant Extracts

Plant samples were purchased from a local market in Selangor, Malaysia. Voucher specimens of *P. nigrum* (047695), *P. betle* (047696) and *G. gnemon* (047698) have been deposited in the Herbarium of University of Malaya. Samples were washed with sterile distilled water and finally rinse with 70% (v/v) ethanol before drying in the oven at 45 °C for three days. Dried samples were grounded to fine powder and soaked sequentially in hexane, chloroform and methanol. The extracts were then filtered through Whatman No. 1 filter paper. Removal of solvents from filtrate was done using a rotary evaporator (EYELA, Tokyo, Japan). Plant extract was dissolved in 100% DMSO (v/v) and were diluted using ultrapure water prior to be used.

### 2.2. Bacterial Strains, Growth Media and Culture Conditions

*P. aeruginosa* PA01 used in this study is from the lab collection while *C. violaceum* CV026 is a double mini-Tn*5* mutant derived from ATCC 31532, Kan^R^, Hg^R^, *cvil*::Tn*5 xyl*E, plus spontaneous Str^R^ AHL biosensor [8]. On top of that, *E. coli* [pSB401] was constructed as a result from *luxRluxl*' (*Photobacterium fischeri* [ATCC7744])::*luxCDABE* (*Photorhabdus luminescens* [ATCC 29999]) fusion, pACYC184-derived, Tet^R^, AHL biosensor while *E. coli* [pSB1075] was derived from *lasRlasl*' (*P. aeruginosa* PAO1)::*luxCDABE* (*Photorhabdus luminescens* [ATCC 29999]) fusion in pUC18 Amp^R^, AHL biosensor [28]. Unless otherwise stated, bacteria were routinely grown in Luria-Bertani (LB) medium (1% w/v NaCl, 1% w/v Tryptone, 0.5% w/v yeast extract) with shaking (220 rpm). *C. violaceum* CV026, were cultured in 28 °C, while *P. aeruginosa* strains at 37 °C. *C. violaceum* CV026 growth medium was supplemented with kanamycin (30 μg/mL) and chloramphenicol (30 μg/mL).

### 2.3. QS Inhibition against *C. violaceum* CV026

Briefly, 15 mL of overnight *C. violaceum* CV026 culture was added to 200 mL of molten LB agar that has been supplemented with C6-HSL(0.25 μg/mL). The *C. violaceum* CV026 agar suspension was poured into Petri dishes. Wells were made using sterile pipette tips once the agar solidified. Plant extract was placed in each well and DMSO (50% v/v) served as the negative control. The Petri dishes were incubated at 28 °C for 24 h. Halo formation on a purple background suggested that the plant extracts exhibited anti-QS. The violacein formed was quantified by incubating *C. violaceum* CV026 (supplemented with C6-HSL, 0.125 μg/mL) with plant crude extract in 96-well plate. The plate was incubated at 28 °C and after 16 h, the 96-well plate was completely dried at 60 °C. Then, DMSO (100 μL) was added onto each well and the 96-well plate was placed in the lab shaker [29]. The reading of the solubilized violacein was taken using a DYNEX MRX Elisa reader (Chantilly, VA, USA) at 590 nm.

### 2.4. Bioluminescence Assay of Biosensors *E. coli* [pSB 401] and *E. coli* [pSB 1075]

AHLs of 0.005 μg/mL [*N*-(3-oxohexanoyl)-L-homoserine lactone] and 0.0125 μg/mL [3-oxo-C12-HSL] were added respectively into overnight culture of *E. coli* [pSB401] and *E. coli* [pSB1075] biosensor cells to induce bioluminescence expression. Then, *E. coli* biosensor cells (230 μL) and plant extract (20 μL) were added into the well of a 96-well microtitre plate. The bioluminescence and OD_495nm_ were determined every 30 min for 24 h using a Tecan luminometer (Infinite M200, Mannerdorf, Switzerland). Expression of bioluminescence was given as relative light unit (RLU)/OD_495nm_ against time [30].

### 2.5. Anti-QS against *P. aeruginosa* PA01 Pyocyanin and Swarming

Overnight culture of *P. aeruginosa* PA01 was diluted to OD_600 nm_ 0.2. Then, plant extract (250 μL) was added to *P. aeruginosa* PA01 (4.75 mL) and incubated at 37 °C for 24 h. The treated culture was extracted with chloroform (3 mL), followed by mixing the chloroform layer with 0.2 M HCl (1 mL). Absorbance of the extracted organic layer was measured using the UV-visible spectrophotometer (UV1601, Shidmazu, Kyoto, Japan) at 520 nm [31]. Swarming agar used in this study consists of glucose (1% w/v), Bacto agar (0.5% w/v), bactopeptone (0.5% w/v) and yeast extract (0.2% w/v). Briefly, solidified swarming agar (10 mL) was overlaid with swarming agar (4.75 mL) supplemented with plant extract (250 μL). Then, overnight culture of *P. aeruginosa* PA01 was inoculated onto the centre of the agar. The plate was incubated for 16 h at 37 °C [21].

### 2.6. Bacterial Growth

Bacterial growth was measured using methods by Hayouni and colleagues with modifications. Briefly, overnight cultures of *P. aeruginosa* PA01, *C. violaceum* CVO26, *E. coli* [pSB 401] and *E. coli* [pSB 1075] were diluted to OD_600nm_ 0.001. Then, the bacteria and plant extracts were placed in a 96-well microtitre plate to make up a final volume of 250 μL in each well. The optical density OD_600nm_ were determined every 30 min for 24 h by a Tecan luminometer (Infinite M200). Bacteria growth was determined by plotting the OD_600nm_ against time [32].

### 2.7. Statistical Analysis

The significance differences between the mean values were tested using ANOVA test (P < 0.05) using GraphPad Prism software. All the assays were performed in triplicate.

## 3. Results and Discussion

*P. nigrum*, *P. betle* and *G. gnemon* used in this study had been used as food sources in the Southeast Asia region for centuries. These plants serve not only as foods, but they are also used as medicinal plants, although little is known about the anti-QS properties of these plants. This study provides a new insight on the anti-QS capabilities of these plant samples.

**Figure 1.**
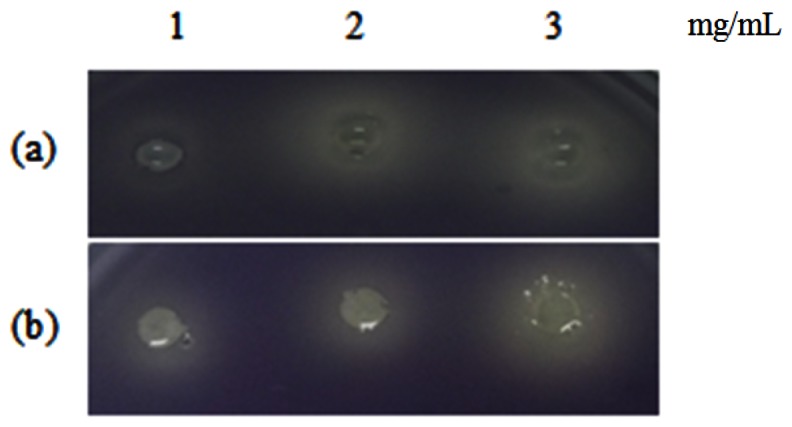
Violacein inhibition by (**a**) *P. nigrum* (chloroform) extract; (**b**) *P. nigrum* (Methanol) extract.

Table 1 shows the summary of the results obtained from this study. Violacein is a purple pigment produced by *C. violaceum*. It is a strong antioxidant and acts by protecting the bacteria membrane against oxidative stress [33]. Three extracts of *P. nigrum* and two extracts of *P. betle* (hexane and chloroform) causes halo formation on the purple background, indicating that these extracts have anti-QS properties against *C. violaceum* CV026. The formation of the halo zone around the well can be observed clearly in Figure 1. Plants extracts have been shown to exhibit anti-QS activity against *C. violaceum* CV026. For instance, *Tremella fuciformis*, vanilla, peas, black olive, bottle brush and graceful sandmat have shown their ability in inhibiting violacein production in previous study [20,34–36]. *E. coli* [pSB 401] and *E. coli* [pSB 1075] produces luminescence in the presence of short chain and long chain AHLs, respectively. Table 1, shows that all the plant samples interrupted response of *E. coli* [pSB 401] to short chain AHLs tested but only handful few samples acted against *E. coli* [pSB 1075]. This could suggest that the plant extracts may interrupt QS that depends on short chain AHLs but not long chain AHLs.

**Figure 2.**
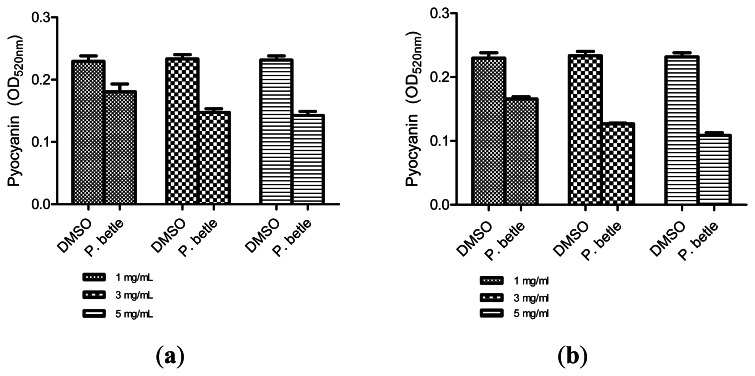
*P. aeruginosa* PA01 pyocyanin formed after addition of (**a**) *P. betle* (hexane extract); (**b**) *P. Betle* (chloroform extract).

**Figure 3.**
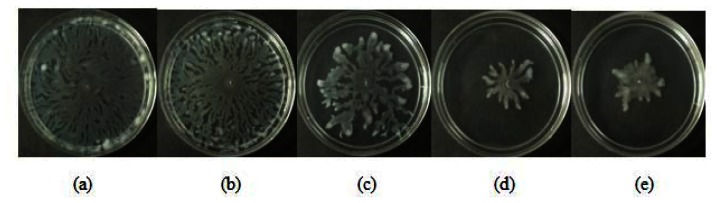
Effect of *P. betle* methanolic extract against *P. aeruginosa* PA01 swarming motility; (**a**) swarming agar with the addition of (**b**) solvent (DMSO 30%); (**c**) *P. betle* methanolic extract (1 mg/mL); (**d**) *P. betle* Methanolic extract (2 mg/mL); (**e**) *P. betle* Methanolic extract (3 mg/mL).

All three extracts of *P. betle* showed significant inhibition against pyocyanin formation by *P. aeruginosa* PA01. Quantified pyocyanin from hexane and chloroform extracts of *P. betle* (Figure 2) decreases with increasing plant extract concentration. Pyocyanin is a secondary metabolite produced by *P. aeruginosa* PA01. Pyocyanin can be found in large quantities in the sputum of cystic fibrosis patients and it disturbs ion transports, ciliary beatings and mucus secretion in the respiratory epithelial cells [37]. Swarming usually occurs on semi-solid agar and this motion enables the cell to colonize the surrounding surfaces [38]. The swarming agar that we had optimized in this study comprised of 0.5% (w/v) agar. Swarming inhibition against *P. aeruginosa* PA01 can be seen in swarming agar that has been seeded with *P. betle* (Methanol) extracts (Figure 3). Extend of inhibition increases as the concentration of plant extract increases. Pyocyanin formation and swarming motility are the virulent phenotypes that are regulated by QS. Compounds from *P. betle* were able to influence the synthesis of these virulent determinants and this makes *P. betle* extracts good candidate for further studies.

**Figure 4.**
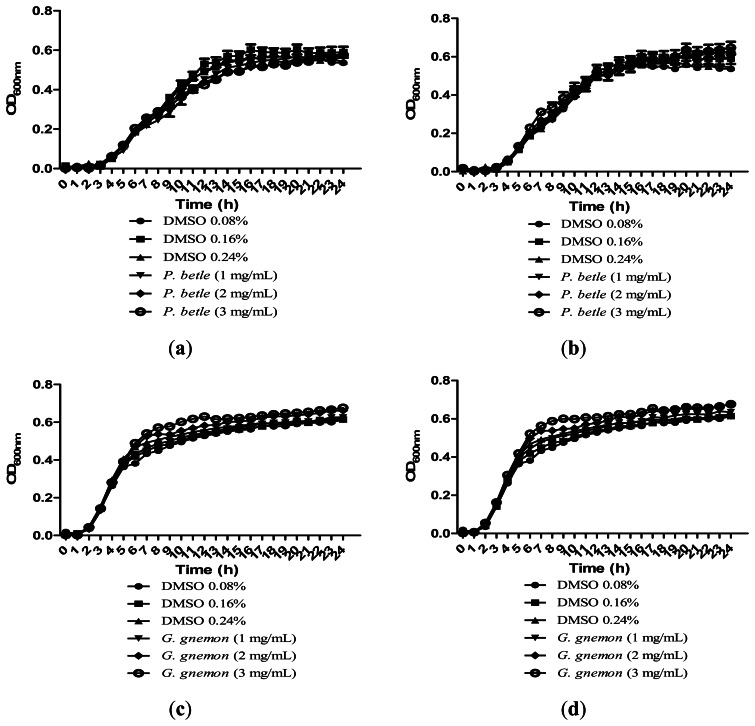
Growth of (**a**) *P. aeruginosa* PA01 in the presence of *P. betle* (hexane extract); (**b**) *P. aeruginosa* PA01 in the presence of *P. betle* (chloroform extract); (**c**) *E. coli* [pSB 1075] in the presence of *G. gnemon* (hexane extract); (**d**) *E. coli* [pSB 1075] in the presence of *G. gnemon* (chloroform extract).

Growth of bacteria was monitored over the course of 24 h to make sure that the inhibitions caused by the plant extracts were solely due to anti-QS and not due to inhibition of bacterial growth. All of the extracts in Figure 4 showed significant inhibition against QS and results obtained after 24 h showed that the plant extracts only have anti-QS properties and did not cause any form of inhibition in the bacterial growth. Same observation was obtained from other plant extracts when cultured together with other bacterial strains. DMSO of final concentration of 0.08%, 0.16% and 0.24% serves as the negative controls at each corresponding concentration.

Consequently, the results obtained from this study also showed that the extracts of *P. betle* have the most effective inhibition against QS as compared to *P. nigrum* and *G. gnemon*. We cannot be sure at which level QS has been modulated by these extracts because the extracts could be competing or disrupting the AHLs binding to the receptors by degradation of AHLs; blocking AHLs from forming AHL-receptor complex; changing the structures of the enzymes that is involved AHLs synthesis. Tertiary plants have developed some form of protective mechanism against bacterial infections through evolution. It is proven through studies which found that chestnut honey, *Terminalia catappa*, Chinese medicinal plants, *Scorzonera sandrasica* and Italian medicinal plants possess anti-QS activity [39–43]. Since they are food sources, the compounds derived will have lower possibility of causing any unwanted reaction in the human system. The samples used in this study can be widely found in Malaysia. Our future goal is to screen for more plants in Malaysia that possess anti-QS.

## 4. Conclusions

Usage of antibiotics has caused pathogenic bacteria to become resistant and poses a global threat to public health. QS provides an alternative solution because by targeting bacterial communication the expression of the virulence phenotype is inhibited. Our data illustrated that these plants possess compounds that can be used to quench QS-mediated virulence determinants. Currently, we are in the process of isolating these compounds using column chromatography and preparative HPLC, and molecular structures will then be determined by mass spectrometry and NMR spectroscopy.

## Figures and Tables

**Table 1.** Results obtained from the assays performed for anti-QS. (√) indicates that the plant extract has anti-QS properties while plant extracts with (-) has no significant inhibition against QS.

Plant samples (solvent extract)	Violacein Quantification Assay	*C. violaceum*Plate Assay	*E. coli* [pSB 401]	*E. coli* [pSB1075]	Pyocyanin Assay	Swarming Assay

*P. nigrum* (hexane)	√	√	√	-	-	-
*P. nigrum* (chloroform)	√	√	√	-	-	-
*P. nigrum* (methanol)	√	√	√	-	-	-
*P. betle* (hexane)	√	√	√	√	√	-
*P. betle* (chloroform)	√	√	√	-	√	-
*P. betle* (methanol)	√	-	√	-	√	√
*G. gnemon* (hexane)	-	-	√	-	-	-
*G. gnemon* (chloroform)	-	-	√	√	√	-
*G. gnemon* (methanol)	-	-	√	√	-	-
